# Alternative Face-on Thin Film Structure of **Pentacene**

**DOI:** 10.1038/s41598-018-37166-6

**Published:** 2019-01-24

**Authors:** Nobutaka Shioya, Richard Murdey, Kazuto Nakao, Hiroyuki Yoshida, Tomoyuki Koganezawa, Kazuo Eda, Takafumi Shimoaka, Takeshi Hasegawa

**Affiliations:** 1 Laboratory of Chemistry for Functionalized Surfaces, Division of Environmental Chemistry, Institute for Chemical Research, Kyoto University, Gokasho, Uji, Kyoto, 611-0011 Japan; 2 Laboratory of Molecular Aggregation Analysis, Division of Multidisciplinary Chemistry, Institute for Chemical Research, Kyoto University, Gokasho, Uji, Kyoto, 611-0011 Japan; 3 Graduate School of Engineering, Chiba University, 1-33 Yayoi-cho, Inage-ku, Chiba, 263-8522 Japan; 4 Molecular Chirality Research Center, Chiba University, 1-33 Yayoi-cho, Inage-ku, Chiba, 263-8522 Japan; 5 Japan Synchrotron Radiation Research Institute, 1-1-1 Kouto, Sayo-cho, Sayo-gun, Hyogo, 679-5198 Japan; 6 Department of Chemistry, Graduate School of Science, Kobe University, 1-1 Rokko-dai, Nada-ku, Kobe, Hyogo, 657-8501 Japan

## Abstract

Pentacene attracts a great deal of attention as a basic material used in organic thin-film transistors for many years. Pentacene is known to form a highly ordered structure in a thin film, in which the molecular long axis aligns perpendicularly to the substrate surface, i.e., end-on orientation. On the other hand, the face-on oriented thin film, where the molecular plane is parallel to the substrate, has never been found on an inert substrate represented by SiO_2_. As a result, the face-on orientation has long been believed to be generated only on specific substrates such as a metal single crystal. In the present study, the face-on orientation grown on a SiO_2_ surface has first been identified by means of visible and infrared p-polarized multiple-angle incidence resolution spectrometry (pMAIRS) together with two-dimensional grazing incidence X-ray diffraction (2D-GIXD). The combination of the multiple techniques readily reveals that the face-on phase is definitely realized as the dominant component. The face-on film is obtained when the film growth is kinetically restricted to be prevented from transforming into the thermodynamically stable structure, i.e., the end-on orientation. This concept is useful for controlling the molecular orientation in general organic semiconductor thin films.

## Introduction

Pentacene is one of the most basic semiconductor materials used in organic thin-film transistors. The aggregation structure, optoelectronic property and their relationship have been attracting keen attention extensively in the field of organic electronics^1–4^. The compound is known to comprise at least three polymorphs, the “thin-film”, “bulk” and “single-crystal” phases^5,6^. If the compound grows on an inert substrate such as SiO_2_, pentacene typically exhibits the “thin-film” phase that is different from its single crystal structure involving both bulk and single-crystal phases. Then, nucleation of the “bulk phase” occurs above a certain critical thickness determined by the deposition condition^1–3^.

The molecular orientation of pentacene is also studied extensively, and in most cases the molecules are known to take the upright standing structure (“end-on” orientation) in a thin film^1–3^. In a vapor-deposited organic film, in general, switching between the end-on and face-on orientations is simply influenced by the two factors: the intermolecular interaction in the film and the interaction between the molecule and the substrate^7–14^. Linear planar molecules represented by pentacene have a strong anisotropy in terms of the intermolecular interaction in a crystal lattice: the intermolecular interaction within the *ab* plane (i.e., the (001) plane) is larger than that along the *c* axis, which makes the (001) plane have the lowest surface energy among the crystal planes^7^. In this case, the (001) surface tends to be oriented parallel to the substrate surface, which is referred later in the discussion of the end-on orientation.

In the case of a “single molecule” (or a small cluster), on the other hand, the face-on stance is generally favored on the substrate surface, since the face-on oriented molecule more positively interacts with the substrate^9,10,12–14^. In this manner, the end-on orientation is mostly driven by the intermolecular interaction; whereas the face-on orientation is induced by the molecule-substrate interaction. For example, the face-on crystallites of pentacene are grown on graphene (and graphite)^15,16^ or metallic substrates^17,18^ whose surface has a strong interaction with the pentacene molecules. Through the consideration of these two extreme cases, both a weak intermolecular interaction and/or positive interaction with a substrate seem necessary to realize the face-on phase.

The intermolecular interaction in a film is strongly influenced by the experimental parameters of the deposition process. The most significant parameter is the substrate temperature, which plays a crucial role for the diffusion kinetics of the molecules on the substrate^3^. Heating the substrate provides sufficient time for nuclear growth, which yields a thermodynamically-stable structure such as the end-on polycrystalline aggregates. Some research groups demonstrate that the end-on orientation phase of diindenoperylene is found when the deposition is carried out at a high substrate temperature (~130 °C), while the face-on one is obtained at a relatively low temperature^10,19^. This gives us an expectation that the low-temperature (LT) film of pentacene also exhibits the face-on phase even on an inert substrate surface. Nevertheless, the structural transition between the end-on and face-on orientations of pentacene on an “inert” substrate has not been observed thus far.

**Figure 1:**
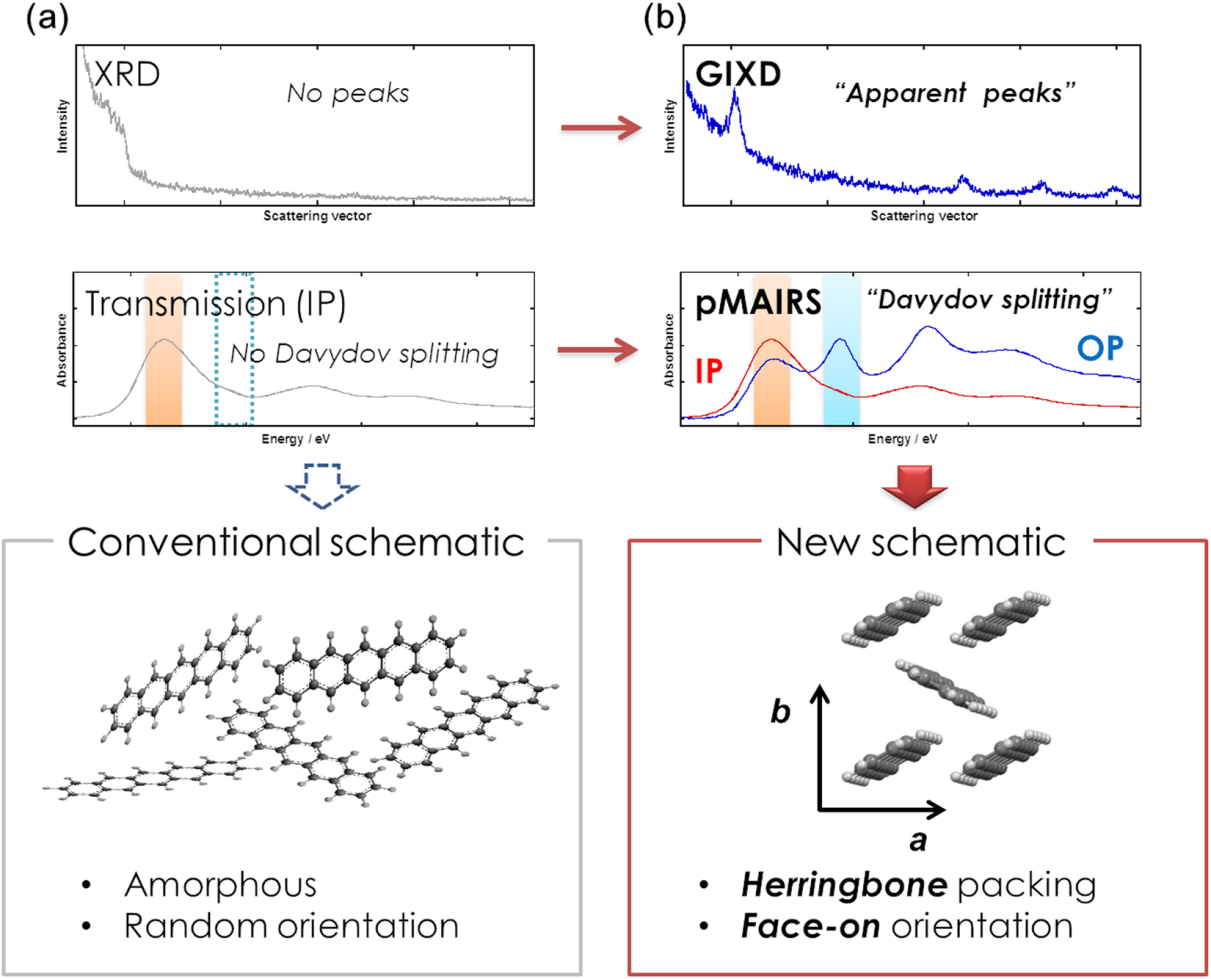
Conventional (**a**) and new **(b**) schematic representations of molecular structure in a pentacene low-temperature thin film revealed by XRD and UV-Vis spectrometry.

In a low-temperature region (≲223 K) of the substrate, pentacene molecules have long been believed to take a randomly oriented amorphous structure (Fig. 1a). Actually, some research groups show that the LT film (followed by storing at room temperature) has no X-ray diffraction (XRD) signals^20–22^, which is supported by no Davydov splitting^23^ in the UV-visible absorption spectrum (Fig. 1a)^24,25^, since the splitting reflects the herringbone crystalline packing of pentacene molecules. These results make us question why pentacene does not exhibit the face-on phase in a thin film, and why the amorphous phase is not transformed into the thin-film phase under ambient temperature.

In the present study, we have very carefully reinvestigated the molecular structure in the pentacene LT film by using a combination of visible (Vis) and infrared (IR) p-polarized multiple-angle incidence resolution spectrometry (pMAIRS)^26–28^. The Vis-pMAIRS spectra give us a strong impression that the conventional normal-incidence transmission spectrum lacks half of the molecular information of the LT film, which misses the Davydov splitting. Through the measurements, we have thus revealed a new intermediate phase with a local minimum potential in the LT film, in which the molecules are oriented in an apparently face-on manner as schematically shown in Fig. 1b. The face-on thin film structure of pentacene is also confirmed by using a surface-sensitive XRD technique of two-dimensional grazing incidence X-ray diffraction (2D-GIXD)^29^. In short, the present study shows that the conventional schematic of the “amorphous” structure should be re-evaluated.

## Results and Discussion

**Figure 2:**
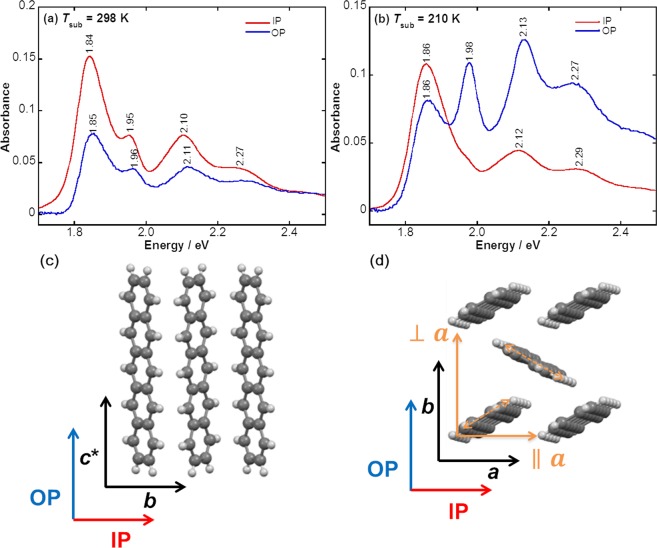
Vis-pMAIRS spectra of pentacene thin films prepared at 298 (**a**) and 210 K (**b**). Two different molecular orientations in pentacene thin films (**c**,**d**).

As mentioned above, the Davydov doublet bands of pentacene are known to be changed into a single band in the UV-Vis “transmission” spectrum as the substrate temperature decreases (Fig. 1a)^24,25^. This spectral change has long been used as evidence to conclude a randomly oriented amorphous structure in the LT film, since Davydov splitting can be correlated with a herringbone crystalline packing of pentacene^30–32^. The anisotropic molecular packing in the LT film, however, cannot be determined only by the transmission measurements that yield only the “in-plane (IP)” component of the transition dipole moment (TDM), which is parallel to the film surface^28^. Both IP and “out-of-plane (OP)” spectra are thus necessary for identifying the anisotropic molecular packing in the LT film. In the present study, Vis-pMAIRS that provides the OP spectrum of a thin film as well as the IP one is first employed for fully monitoring Davydov splitting of an organic thin film (Fig. 2).

The film grown at room temperature (denoted as “RT-film”) exhibits the Davydov components at 1.84 and 1.95 eV in both IP and OP spectra in Fig. 2a. The lower energy component is known to have the TDM along the *a* axis (||*a*), and the higher one has that nearly along the *b* axis (⊥*a*)^30–32^, where both moments are orthogonal to the c* axis (Fig. 2d). As found in Fig. 2a, both Davydov peaks in the IP spectrum appear stronger than that in the OP spectrum, which implies that both *a* and *b* axes, i.e., the *ab* plane is nearly parallel to the substrate surface. In other words, the c* axis is nearly vertical to the substrate surface, confirming the conventionally agreed end-on stance where the molecular long axis is nearly parallel to the c* axis (Fig. 2c). Quantitative orientation analysis on the “Vis”-pMAIRS spectra, however, is not performed in the present paper, which needs another correction factor set^33^. With the qualitative discussion, fortunately, the essentially important experimental fact has readily been revealed.

To our surprise, a significant difference of the spectral shape is apparently found for the film grown at 210 K (“LT-film”). As indicated in Fig. 2b, the “singlet” band at 1.86 eV is observed dominantly in the IP spectrum corresponding to the conventional transmission one, which reproduces the former studies^24,25^. This annihilation of Davydov splitting in the “IP” spectrum gives us an impression that the molecules do not keep the herringbone packing structure in the LT-film. This conventional interpretation is largely revised in the present study, however, because another Davydov component appears selectively in the “OP” spectrum at 1.98 eV (Fig. 2b). In other words, Davydov splitting has long been missed thus far because only the IP spectrum is discussed by the measurement of the transmission spectrum (Fig. 1). The Davydov components at 1.86 and 1.98 eV are thus individually detected in the IP and OP spectra, respectively, which indicates that the herringbone packing structure of pentacene does exist in the LT-film. This is a great benefit of using the Vis-pMAIRS technique.

Interestingly, the IP and OP spectra of the LT-film are nearly identical to the previously reported polarized spectra of a single crystal along the *a*- and *b*-axes^30–32^, respectively. This result means that the *a* axis is oriented parallel to the substrate surface, i.e., the IP direction, while the *b* axis has an upright stance as shown in Fig. 2d. On considering the TDM of the band at 1.98 eV along the *b* axis (⊥*a*), in effect, the selective appearance of this band in the OP spectrum agrees with the schematic of the face-on structure (Fig. 2d). In this situation, the *a* axis must be directed parallel to the IP direction (Fig. 2d). This is confirmed by the relatively strong IP band at 1.86 eV (||*a*) in Fig. 2b. Note that the weak band at 1.86 eV remains in the OP spectrum reflecting misoriented crystallites of pentacene in the LT-flm as discussed in the XRD section. In this manner, the LT-film has definitely been determined to have the face-on orientation as the schematic in Fig. 2d.

The discussion on the Vis-pMAIRS results can readily be supported by IR-pMAIRS measurements, which are powerful for revealing the molecular orientation of each normal mode as well as the molecular packing^34–36^. The most important bands are the C–H out-of-plane deformation vibration (γ(C–H)) bands of an aromatic ring that appear typically in a region less than 1000 cm^−1^. On the surface selection rule (SSR) of pMAIRS^27^, the intensity ratio of the IP band to the OP one reveals the ring orientation^34–36^, since the IP and OP spectra have a common ordinate scale^33^.

**Figure 3:**
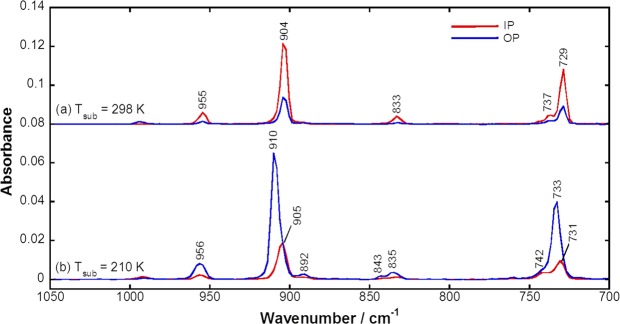
IR-pMAIRS spectra of pentacene thin films prepared at 298 (**a**) and 210 K (**b**).

Fig. 3 shows IR-pMAIRS spectra of pentacene thin films in the γ(C–H) region. All the bands appeared in Fig. 3 are assigned to the γ(C–H) modes^37^ of the coupled oscillations over the fused benzene rings except for the weak band at ~990 cm^−1^. The IP bands (red) are stronger than the OP one (blue) for the RT-film (Fig. 3a), while the interrelation is overturned for the LT-film (Fig. 3b). Considering SSR of pMAIRS, the RT- and LT-films are readily categorized into the end-on (or edge-on) and face-on orientations^34–36^, respectively, which strongly supports the Vis-pMAIRS discussion.

Another important point is that the “OP” bands of the LT-film exhibit a band shift to a higher position than that of the RT-film as typically found for the band at 910 cm^−1^ in Fig. 3b. The band position is influenced by the molecular stacking and packing through the intermolecular interaction. The polymorphs can thus be identified from the band position^15,34,36,38–40^. By referring to former studies^15,39,40^, the bands of the RT-film are assigned to the thin-film phase, while that of the LT-film suggests another polymorph that is discriminated from the generally accepted phases, i.e., the thin-film (904 cm^−1^)^15,39,40^ and bulk (906 cm^−1^)^40^ phases. This is confirmed by the interplanar spacing of the (001) surface (*d*_001_) calculated from the GIXD pattern with an in-plane geometry (see Fig. S1 in the Supplementary Information).

Of note is that, for the LT-film (Fig. 3b), the “IP” bands appear at a lower-wavenumber position than the OP bands, which is apparently found for the band at 905 cm^−1^. According to our previous study^35^, in a face-on/edge-on mixed film, the γ(C–H) band position in the IP spectrum is mainly attributed to the edge-on (or end-on) component, while the OP band position is determined dominantly by the face-on one. The lower-wavenumber position (905 cm^−1^) in the IP spectrum should thus correspond to another component of the thin-film (end-on) phase (904 cm^−1^; Fig. 3a) rather than the face-on one. In other words, the end-on component coexists as a minor component in the LT-film. Also, the pMAIRS spectra of the LT-film are impervious to the film thickness (Fig. S2), which indicates that the two phases are nucleated competitively on the substrate surface. Note that the pMAIRS spectra provide average orientation information in a thin film. The orientation distribution of each crystallite cannot be determined from the spectra. To reveal the details of crystalline distributions in the pentacene films, 2D-GIXD measurements are employed as follows.

**Figure 4:**
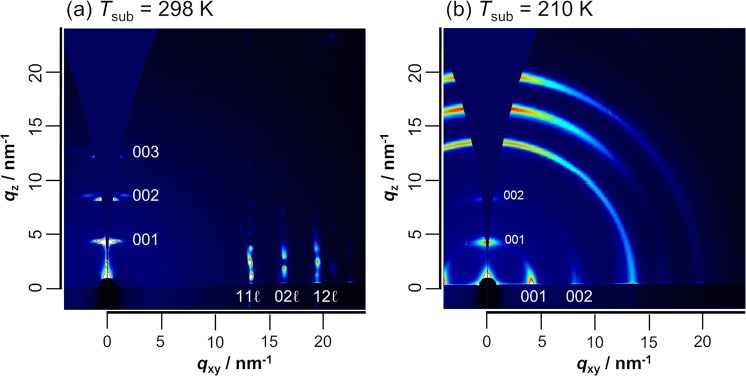
2D-GIXD patterns of pentacene thin films prepared at 298 (**a**) and 210 K (**b**).

As found in Fig. 4a, the RT-film has pronounced peaks due to the (00ℓ) reflections along the out-of-plane component of the scattering vector (*q*_*z*_), which are induced by the thin-film and bulk phases^41,42^. This means that the c* axis is nearly vertical to the substrate surface, corresponding to the end-on stance (Fig. 2c). Other diffraction peaks (11ℓ, 02ℓ and 12ℓ with ℓ = 0 or ± 1) are observed nearby the in-plane component (*q*_*xy*_). This is a well-known typical 2D-GIXD pattern of pentacene thin films^41–46^.

As expected from the pMAIRS results, on the other hand, the LT-film yields a reversed result: the 00ℓ peaks appear almost along the *q*_*xy*_ direction as found in Fig. 4b. This overturned result indicates a drastic orientation change of the c* axis. The LT film is found to have a nearly parallel orientation of the c* axis to the substrate surface, that is, the molecule is not categorized into the end-on orientation. Other reflections in the region more than *q* = 10 nm^−1^, on the other hand, appear strongly nearby the *q*_*z*_ axis as a partial ring (Fig. 4b), which means that the other two axes are not correlated with the random orientation, but they have a relatively broad orientation distribution as compared to that of the *c* axis. The difference of the orientational distribution suggests that the face-on aggregates are interpreted to have a liquid crystal-like structure, which agrees with a theoretical prediction that the face-on orientation is generated when the crystal growth is kinetically restricted. From the GIXD pattern, however, the crystal orientation cannot be determined accurately due to the overlap of the diffraction peaks. Of another note is that the end-on phase still coexists in the LT-film as found by the relatively weak 00ℓ peaks appeared on the *q*_*z*_ axis.

It should additionally be mentioned that the GIXD technique enables us to analyze a thin film with very high sensitivity as compared to the conventional specular reflection method^29^. The LT-film shows no apparent diffraction peaks in the specular diffraction pattern (Fig. S3a); whereas the GIXD patterns involve some weak but apparent signals in Fig. S3b,c. In total, the LT-film is found to have a low crystallinity requiring a surface-sensitive technique, but it *cannot* be categorized into amorphous. The face-on structure generated in the LT-film should be considered as an intermediate phase between the thin-film and the amorphous phases. Thus, the face-on phase is kinetically favored when the surface diffusion of molecules is restricted on a cold substrate. These results experimentally support a former study using molecular dynamics (MD) simulations^12^.

It is noteworthy that the face-on orientation is not switched to the end-on one even after storing at ambient temperature (for at least a month), although the end-on aggregates are thermodynamically advantageous. This means that the energetic barrier between the face-on and end-on phases, i.e., the reorientation barrier, exceeds the thermal energy.

In this manner, the face-on phase in pentacene thin films has been identified for the first time by means of Vis-pMAIRS, IR-pMAIRS and 2D-GIXD. We believe that the face-on phase is a general property of organic semiconductors^47–49^ having an anisotropic molecular shape, not limited to pentacene.

## Methods

### Sample preparation

Vapor-deposited films of pentacene were prepared on a contamination-free surface of a silicon substrate (SiO_2_) after an ozone-treatment under a base pressure of 1.0 × 10^−4^ Pa. The film thickness was ~50 nm, and the average deposition rate was ~60 nm min^−1^ for every sample. The substrate was cooled by liquid nitrogen, and then the as-deposited film was stored overnight in vacuo at room temperature (~298 K). The temperature, *T*_sub_, was measured by a thermocouple directly contacted on the substrate surface. The optimal temperature region to obtain the face-on oriented film was found to be 200 K < *T*_sub_ < 250 K. In this paper, the film grown at ~210 K (LT-film) is compared to that at room temperature (RT-film). The films were characterized using Vis-pMAIRS, IR-pMAIRS and 2D-GIXD. For the Vis-pMAIRS measurements, the films with a thickness of 20 nm were prepared on a quartz substrate.

### Vis-pMAIRS measurements

The Vis-pMAIRS measurements were carried out on an Otsuka Electronics (Tokyo, Japan) MCPD 7000 UV-visible spectrophotometer^28^. The spectra were recorded with an ultrahigh-sensitive CCD multichannel photodiode array detector. The angle of incidence was varied from 8° to 38° in 6° steps. The accumulation number was 100.

### IR-pMAIRS measurements

The IR-pMAIRS spectra were obtained by a Thermo Fischer Scientific (Madison, WI) Nicolet 6700 FT-IR spectrometer equipped with a Thermo Fischer Scientific (Yokohama, Japan) automatic MAIRS equipment (TN 10-1500). The p-polarized IR ray was generated through a PIKE Technologies (Madison, WI) manual polarizer (090-1500). The IR ray was detected by a liquid-nitrogen-cooled MCT detector. The angle of incidence was changed from 9° through 44° by 5° steps. The number of accumulation of the interferogram was 500 for each angle of incidence.

### XRD measurements

The 2D-GIXD patterns were measured by a HUBER (Rimsting, Germany) diffractometer at the BL46XU beamline of SPring-8 (Hyogo, Japan). The X-ray was selected to have the energy of 12.39 keV (λ = 0.1 nm), and the reflected ray from the sample surface was detected by a 2D image detector (Pilatus 300 K). The incident angle of X-ray was fixed at 0.12° from the surface parallel. The 1D-XRD measurements were performed on a Rigaku (Tokyo, Japan) Superlab^50^ X-ray diffractometer using Cu Kα radiation (λ = 0.154 nm) generated at 40 kV and 30 mA. The GIXD patterns were measured with an in-plane and out-of-plane geometries^34^.

## Supplementary information

Supplementary Information
