# Supplementary material for: Alternative Face-on Thin Film Structure of Pentacene
Source: Sci Rep. 2019 Jan 24;9:579. doi: 10.1038/s41598-018-37166-6 (PMC6345764; doi:10.1038/s41598-018-37166-6)
Supplement: Supplementary file 1 — Supplementary Information [file 41598_2018_37166_MOESM1_ESM.docx]

# Supplementary Information

**Alternative Face-on Thin Film Structure of Pentacene**

Nobutaka Shioya^1^, Richard Murdey^2^, Kazuto Nakao^2^, Hiroyuki Yoshida^3,4^, Tomoyuki Koganezawa^5^, Kazuo Eda^6^, Takafumi Shimoaka^1^, and Takeshi Hasegawa^1,*^

^1^Laboratory of Chemistry for Functionalized Surfaces, Division of Environmental Chemistry, Institute for Chemical Research, Kyoto University, Gokasho, Uji, Kyoto 611-0011, Japan

^2^Laboratory of Molecular Aggregation Analysis, Division of Multidisciplinary Chemistry, Institute for Chemical Research, Kyoto University, Gokasho, Uji, Kyoto 611-0011, Japan

^3^Graduate School of Engineering and ^4^Molecular Chirality Research Center, Chiba University, 1-33 Yayoi-cho, Inage-ku, Chiba 263-8522, Japan

^5^Japan Synchrotron Radiation Research Institute, 1-1-1 Kouto, Sayo-cho, Sayo-gun, Hyogo 679-5198, Japan

^6^Department of Chemistry, Graduate School of Science, Kobe University, 1-1 Rokko-dai, Nada-ku, Kobe, Hyogo 657-8501, Japan

*htakeshi@scl.kyoto-u.ac.jp


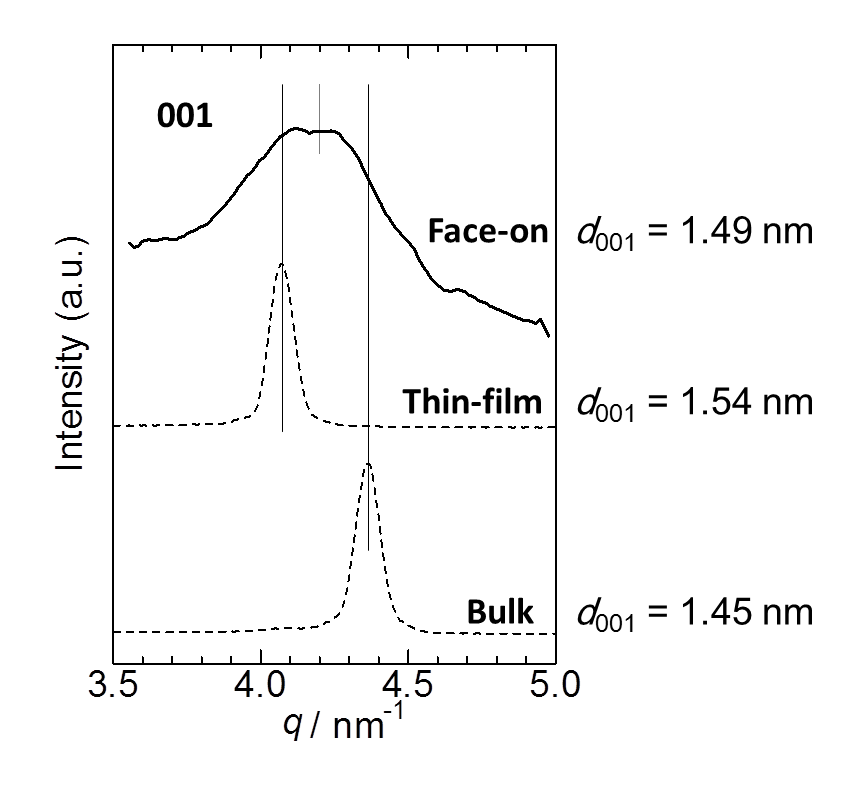


Fig. S1 Magnified GIXD-IP pattern of the 001 peak region in a pentacene film prepared at 210 K (solid line).

Fig. S2 IR-pMAIRS spectra of pentacene thin films with thickness of 20 nm (a) and 50nm (b) prepared at 210 K.


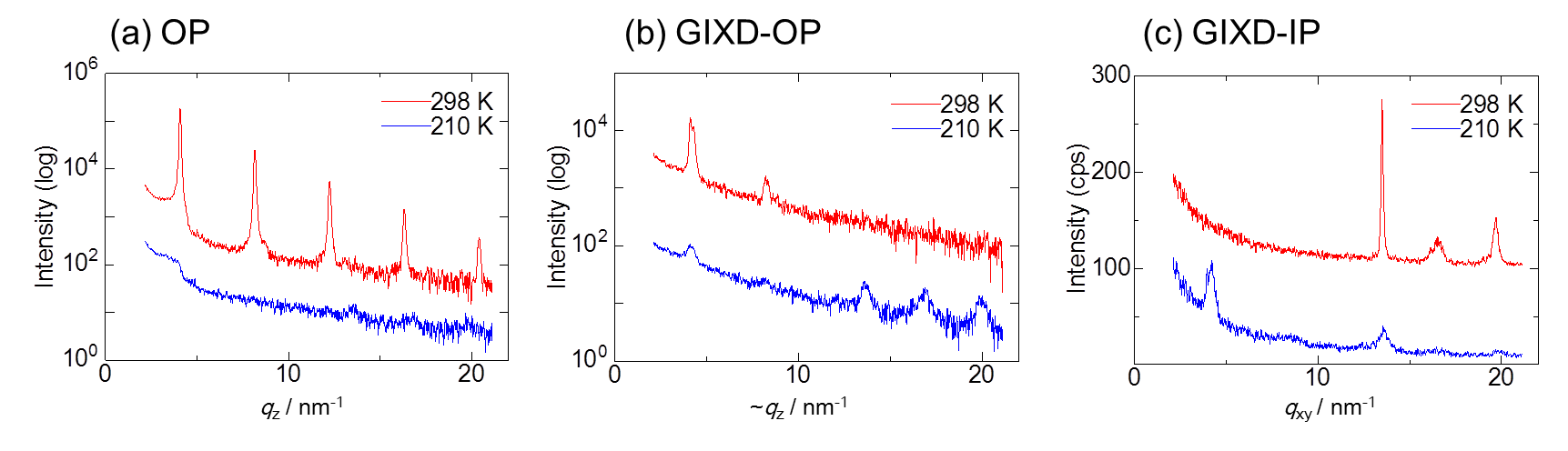


Fig. S3 XRD patterns measured with a symmetric out-of-plane geometry (a), GIXD-OP (b) and -IP (c) patterns of pentacene films prepared at 298 and 210 K.
